# Gender Differences among Sardinians with Alcohol Use Disorder

**DOI:** 10.3390/jcm10204688

**Published:** 2021-10-13

**Authors:** Roberta Agabio, Claudia Pisanu, Luigi Minerba, Gian Luigi Gessa, Flavia Franconi

**Affiliations:** 1 Department of Biomedical Sciences, Section of Neuroscience and Clinical Pharmacology, University of Cagliari, I-09042 Monserrato, CA, Italy; claudia.pisanu@unica.it (C.P.); lgessa@unica.it (G.L.G.); 2 Department of Medical Sciences and Public Health, University of Cagliari, I-09042 Monserrato, CA, Italy; lminerba@gmail.com; 3 Neuroscience Institute, National Research Council of Italy, Section of Cagliari, I-09042 Monserrato, CA, Italy; 4 Laboratory of Sex-Gender Medicine, National Institute of Biostructures and Biosystems, I-07100 Sassari, SAR, Italy; franconi.flavia@gmail.com

**Keywords:** alcohol use disorder, gender differences, Sardinia, birth cohort effects, access to treatment, women’s needs

## Abstract

Sardinia is an Italian island in the Mediterranean characterized by secular isolation and the singular genetic characteristics of its inhabitants. Findings obtained in populations with diverse genetic make-up and cultural background indicate gender differences and/or similarities in drinking characteristics of patients with alcohol use disorder (AUD). Knowledge of these characteristics in AUD patients is useful to improve access to treatments. This paper investigated the drinking characteristics of 66 female and 282 male outpatients with AUD, born from 1937 to 1991, living in Sardinia, and compared their characteristics with those of AUD patients living in other countries. Most Sardinian patients were men, approximately 3 years younger than women; women consumed lower amounts of alcohol than men but did not differ from men in the severity of AUD. Men were more often single than women, while a higher proportion of women reported that their mother or spouse was affected by AUD. Anxiety and depression were more prevalent among women while a higher proportion of men were affected by substance use disorders. Women were older than men at the age of first drink, regular drinking, and onset of AUD, and progressed faster than men from regular use to AUD onset. Women did not differ from men in age at first request for care, and in the lapse from AUD onset to first request for care. Women and men waited for more than 8 and 9 years, respectively, before receiving medical treatment. Gender differences progressively decreased among younger patients. Although the scarce number of women in some cohorts limits the strength of these findings, drinking characteristics of Sardinian patients did not vary significantly from those of AUD patients living in other countries. These results suggest that the number of Sardinian women with AUD is increasing and services for treatment of AUD should (a) consider women’s specific needs, and (b) realize effective policies to reduce latency prior to accessing medical treatment for both men and women with AUD.

## 1. Introduction 

A series of gender (related to psychosocial and cultural issues) and sex differences (related to biological issues) have been described in the drinking characteristics of patients with alcohol use disorder (AUD) [1,2]. Differences in drinking patterns and genetic differences in alcohol metabolizing enzymes have been described among diverse ethnicities [3,4]. The majority of studies on drinking characteristics of patients with AUD have been performed largely in Northern countries [5,6]. Only a very few studies have been performed in Sardinia, a Mediterranean island off the western coast of Italy, characterized by secular isolation and a peculiar genetic make-up of its inhabitants [7]. For example, Sardinia boasts a high incidence of certain autoimmune diseases such as type 1 diabetes [8], and one of the highest concentrations of centenarians in the world [9]. Therefore, the inhabitants of Sardinia have been extensively studied and the results of these investigations indicated that sex-biased processes have substantially impacted the genetic history of Sardinians [8]. Additionally, Sardinia has a low socioeconomic status, with a gross domestic product equal to 70% of the European average, and a low level of education, attested to by a low number of university graduates [10]. The latter aspects are important due to the fact that particularly in low- and middle-income countries, the impact of gender diverges between countries and regions [11,12,13], with intraregional variations being smaller [11]. A higher convergency between men and women in drinking characteristics has been documented in countries with a lower gender gap [14,15,16,17,18], such as the United States [5,6,19,20,21,22,23,24,25,26,27,28,29,30,31,32,33], Canada [34], Germany [35,36], UK [37], and Australia [38].

The most frequently observed gender differences in drinking characteristics consist of the lower rates of women who drink compared with men, women who start drinking later and drink less than men, and in the less frequent occurrence of AUD in women compared with men [39]. In addition, women tend to progress faster to problematic use or AUD, displaying more and earlier-onset negative consequences of alcohol consumption which occur on consumption of lower amounts of alcohol [1,2]. Finally, women are at risk for specific alcohol-related consequences such as fetal alcohol syndrome in their children [40] and breast cancer [41], although less than 20% of women are aware that alcohol consumption is a dose-dependent risk factor for breast cancer [42,43]. Worldwide however, gender differences observed in drinking characteristics among recent birth cohorts have narrowed progressively as women’s drinking patterns have become more similar to those of men [15,16,18,44,45,46,47,48,49,50,51,52]. 

Notably, most AUD individuals never gain access to treatment [53,54,55,56,57,58,59], especially in low and lower-middle-income countries [57]. Numerous non-gender specific barriers to AUD treatment are present, including the consideration that people with AUD are strong enough to manage the disease alone, viewing the disease as being less serious than it is and hoping that it may improve by itself, a lack of awareness of the availability of effective treatments, and stigma (being too embarrassed to discuss with others) [54]. Women-specific barriers are also observed, including feelings of guilt, shame, and responsibility towards their children [60,61]. Furthermore, lower rates of women than men receive AUD treatment [55,62], with pregnant women being less likely to receive AUD treatment than non-pregnant women [63]. 

This paper was keen to evaluate whether gender differences in the drinking characteristics of AUD patients observed in other countries or regions differed from those of AUD patients living in Sardinia, Italy. Accordingly, the present study aimed to (i) investigate differences and/or similarities between drinking characteristics of women and men in a sample of Sardinian AUD patients, (ii) investigate cohort effects in gender differences and/or similarities in drinking characteristics, stratifying the sample into cohorts according to year of birth, and (iii) compare the characteristics of Sardinian AUD patients with those of similar samples of AUD patients living in other countries or regions. 

## 2. Methods

### 2.1. Authorization and Selection of Participants

The study was conducted in a large area in Sardinia, Italy with approximately 550,000 adult inhabitants and four public healthcare facilities that provide outpatient treatment for AUD. This area was selected to recruit patients with heterogeneous socioeconomic backgrounds, from rural, urban, and metropolitan areas. The study was approved by the four local health authorities (LHA) that provided the healthcare facilities (LHA 1: Authorization n. 945 (28 February 2011); LHA 3: n. 1131 (27 October 2011); LHA 6: n. 35 (20 January 2012); LHA 7: n. 85 (21 April 2011)). 

The number of subjects enrolled reflected the prevalence of adult outpatients among public services in Italy [64]. Briefly, according to the national report by the Italian Ministry of Health, in 2011, approximately 58,000 AUD patients were in alcohol treatment programs in Italy (approximately 60,000,000 inhabitants; corresponding to approximately 0.1% of the general population) [64]. According to a regional report, in 2007, approximately 1900 AUD patients were in alcohol treatment programs in Sardinia (approximately 1,600,000 inhabitants; approximately 0.1% of general population) [65]. 

### 2.2. Data Collection

Information was collected by four psychologists during face-to-face interviews conducted between 2011 and 2012. The psychologists were experienced in the treatment of AUD and trained to minimize differences in conducting the interviews. Eligible interview candidates were selected by examining medical records. Subjects were considered eligible if they were 18 years or older, met DSM-IV diagnostic criteria for alcohol dependence and had been in AUD treatment for at least 4 months. Exclusion criteria were lack of knowledge of the Italian language and presence of a number of severe comorbid mental or psychiatric disorders that would prevent understanding during the interview. Eligible participants were invited to take part in the study just before their visit to the doctor or were contacted by telephone to schedule an interview. Participants were free to take part in the study, were assured that their answers would be kept strictly confidential and were not paid for participation. Prior to the interviews, individuals signed an informed consent form and were informed about the size of a standard drink unit (approximately 12 g of alcohol). To describe drinking variables (see below), participants were required to think about a typical month before treatment using the “timeline-follow back”. Each interview lasted approximately one hour.

#### Data Collected

The questionnaire contained the following sections: (a) presentation section (focusing on current and/or previous medical treatments); (b) demographic section; (c) socio-economic section; (d) family history section; (e) diagnosis section (including diagnostic criteria for alcohol dependence and alcohol abuse according to the DSM-IV-TR); and (f) alcohol consumption section and smoking characteristics. This section investigated the following drinking characteristics: (i) age at first drink; (ii) age at onset of regular drinking; (iii) age at onset of AUD; (iv) age at first request for medical care; (v) comorbid physical or psychiatric diseases; (vi) number of drinks per drinking day; (vii) number of drunk days; (viii) number of hospital admissions due to alcohol consumption; (ix) number of suicide attempts; and (x) smoking characteristics. From these drinking characteristics two different lapses (in years) were calculated: (xi) lapse from regular use to AUD (by subtracting age at onset of regular drinking from age of AUD onset); and (xii) lapse from AUD to first treatment (by subtracting age at onset of AUD from age at first request for care). Finally, (xiii) the mean number of DSM-IV-TR positive criteria for alcohol dependence was used to establish the severity of AUD (3: mild; 4–5: moderate; 6–7: severe). 

For the third aim, data were obtained by searching the published medical literature in Medline (PubMed) until May 2021. The search was limited to humans and English language. The search terms used included alcoholism or AUD or alcohol dependence AND treatment. Studies not providing data for both men and women were excluded.

### 2.3. Calculation

Descriptive statistics representing mean, standard deviation (mean (SD)), and percentage were calculated. Normality of distribution was assessed using skewness and kurtosis. Drinking characteristics were first analyzed for the entire sample and then on division of the sample into different cohorts according to the year of birth of participants. Ten-year cohorts were used, with the exception of the first cohort (that also included the few participants born before 1943) and the last cohort (that included the few participants born after 1984). Globally, the four cohorts selected were as follows: cohort A (from 1937 to 1953); cohort B (from 1954 to 1963), cohort C (from 1964 to 1973), and cohort D (from 1974 to 1991). Data of Sardinian AUD patients divided into birth cohorts were provided as mean and 95% confidence interval (CI). Differences (Δ) were expressed in values Δ < 0.0 when men had lower means compared with women, and in values Δ > 0.0 when men had higher means compared with women. Differences between independent samples (males vs. females) were assessed using Fisher’s exact test for categorical variables or two-tailed Student t and Mann–Whitney U tests for quantitative normally-distributed or not normally-distributed variables, respectively. 

The effect of birth cohort, gender and their interaction on drinking milestones was tested using a univariate general linear model with the drinking milestone as the dependent variable, and gender, cohort and their interaction as fixed factors. Analyses were adjusted for demographic and clinical variables found to differ significantly between men and women. Adjusting factors included age at interview, civil status, having a mother with AUD, comorbidity with anxiety, depression, other substance use disorders, and suicidal behavior. Having a spouse with AUD was not included as an adjusting factor in order not to exclude participants without a spouse from these analyses. Analyses were performed using SPSS version 20 (IBM, Armonk, NY, USA).

## 3. Results

### 3.1. Selection of Participants

**Figure 1:**
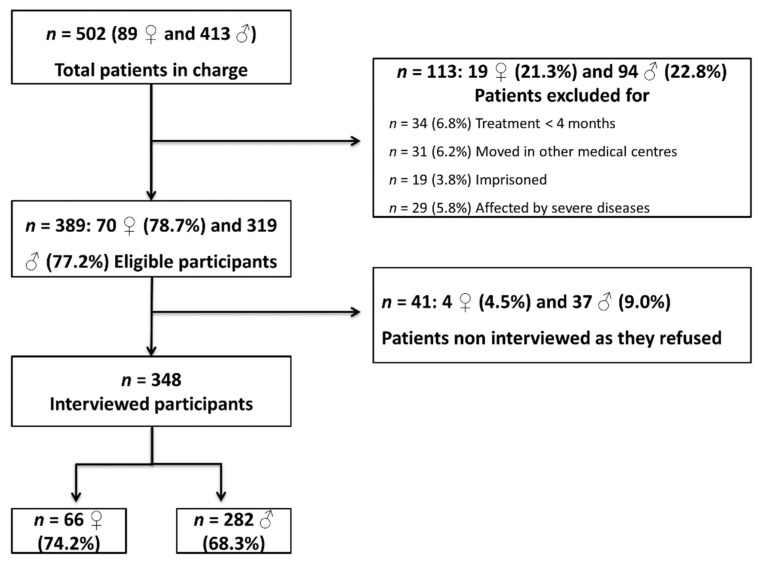
Flow chart indicating the selection process of the study’s sample of Sardinian outpatients with alcohol use disorder. ♀: women; ♂: men.

Examination of medical records revealed that 502 patients were in the care of the four public healthcare facilities in the area selected (approximately 550,000 adult inhabitants; corresponding to approximately 0.1% of the general population). Among these, 113 (22.5%) were excluded for different reasons (e.g., because affected by severe diseases or imprisoned). As shown in Figure 1, 41 of 389 eligible participants were not interviewed because they refused to take part in the study, leaving 348 patients as the sample. Interviewed participants did not differ from those who were not interviewed in sex and gender composition, age, marital status, education or employment, and treatment received (data not shown). 

### 3.2. Gender Differences and Birth Cohort Effects in Drinking Characteristics of AUD Sardinian Patients 

#### 3.2.1. Main Characteristics 

Table 1 shows the main characteristics of the entire sample of AUD Sardinian patients. In Table 2, these characteristics are analyzed dividing the sample into cohorts according to year of birth. In Table 3, the characteristics of the entire sample of AUD Sardinian patients are compared with those of AUD patients living in other countries. As shown in Table 1, the majority of patients were men approximately 3 years younger than women. Women consumed lower amounts of alcohol than men but did not differ from men in the severity of AUD. The men/women ratio (4.3 men: 1 woman), and mean ages of the participants resulted as similar to those encountered among patients in alcohol treatment programs in Italy [64]. In 2011, the men/women ratio in alcohol treatment programs in Italy was 3.7 and mean age was 46.9 and 44.9 years, for women and men, respectively [64]. Conversely, in other studies, women with AUD were either younger than men (see Table 3; [27]) or of the same age [30,36,66,67]. In other studies, no differences were detected as age was part of the matching procedure [37,38,68]. Men were more often single than women, while a higher proportion of women reported that their mother or spouse was affected by AUD. Anxiety and depression were more prevalent among women while a higher proportion of men were affected by substance use disorders. No significant differences were found in other characteristics. Similar findings were reported by other studies (see Table 3).

#### 3.2.2. Age at First Drink

Analyzing the entire sample, women were older than men at the age of first drink (see Table 1) and in cohorts B (years of birth 1954–1963) and C (years of birth 1964–1973) (see Table 2). The same difference was observed by studies conducted in other countries [27,29,36,38,66,67,68] and by a US national survey [6] (see Table 3). The mean age of women progressively decreased from cohorts B to D (years of birth 1974–1991) while the mean age of men remained approximately the same from cohorts B to D. The differences between men and women progressively decreased from cohorts B to D. The model, including sex, cohort and their interaction, significantly predicted age at first drink (F = 3.42, p of the model = 0.002), with a significant contribution of sex (F = 5.08, *p =* 0.025). These results remained significant in the model adjusted for age at interview, civil status, family history, and comorbidities. In the adjusted model (F = 2.64, p pf the model = 0.001), a significant contribution of sex (F = 4.39, *p =* 0.037), cohort (F = 2.80, *p =* 0.040), their interaction (F = 2.78, *p =* 0.041), and age at interview (F = 6.56, *p =* 0.011) were observed, while all the other terms were not significant. 

#### 3.2.3. Age at Onset of Regular Drinking

Women started regular drinking later than men both in the entire sample (see Table 1) and in cohorts B and C (see Table 2), while they tended to be older in cohorts A (years of birth 1937–1953) and D (1974–1991). The differences progressively decreased from cohorts A to D. The mean ages of both men and women progressively decreased from cohorts A to D. The model including gender, cohort and their interaction, significantly predicted regular alcohol use (F = 8.13, *p* < 0.001), with a significant contribution of cohort (F = 3.55, *p =* 0.025) and gender (F = 19.57, *p* < 0.001). The model remained significant after adjusting for demographic or clinical variables showing significant gender differences (F = 4.56, *p* < 0.001). In the adjusted model, gender (F = 16.28, *p* < 0.001) and age at interview (F = 4.79, *p =* 0.029) provided a significant contribution, while all other terms were not significant. These results may be unreliable due to the scarce number of women in cohorts A and D (13 and 4, respectively). However, similar findings of the age at onset of regular drinking were found in other countries (see Table 3; [30,31,32,36,38,68]) and by a study that utilized data from two national US surveys [5]. Conversely, other studies found no gender differences in age at regular drinking [23,27].

#### 3.2.4. Age at Onset of AUD

Sardinian women were older than men at the age of onset of AUD in the entire sample and in cohort A. This difference progressively decreased from cohorts A to C. The mean ages of both men and women progressively decreased from cohorts A to D. The model including gender, cohort and their interaction significantly predicted age at onset of AUD (F = 17.36, *p* < 0.001). Birth cohort gave a highly significant contribution to this model (F = 24.16, *p* < 0.001), and gender and birth cohort significantly interacted (F = 2.64, *p =* 0.049). In the model adjusted for demographic or clinical variables showing significant gender differences (F = 10.49, model *p* < 0.001), the interaction between sex and birth cohort was still significant (F = 3.46, *p =* 0.017). In addition, age at interview (F = 6.02, *p =* 0.015), and civil status (F = 3.75, *p =* 0.025) provided a significant contribution to the model, while all other terms were not significant. The same difference in the age of AUD onset was observed in other countries (see Table 3; [24,32,36,66,68]). Conversely, no differences were found by other studies [6,23,27,29,30,67].

#### 3.2.5. Age at First Request for Care

No differences were found in age at first request for care in the entire sample and in any cohort evaluated. The mean ages of both men and women progressively decreased from cohorts A to D. The model including gender, cohort and their interaction significantly predicted age at first request for care (F = 72.63, *p* < 0.001), with a significant contribution of cohort (F = 92.01, *p* < 0.001). In the model adjusted for demographic or clinical variables showing significant gender differences (F = 43.53 model *p* < 0.001), the interaction between gender and birth cohort remained significant (F = 3.21, *p =* 0.023). In addition, age at interview (F = 45.32, *p* < 0.001) and comorbidity with anxiety (F = 4.82, *p =* 0.029) provided a significant contribution to the model. Other studies found no differences in age at first request for care (see Table 3; [29,31,36,66,67]) while yet others observed how women were younger than men at this drinking milestone [27,32,34].

#### 3.2.6. Lapse from Regular Use to AUD Onset

Women progressed faster than men in the entire sample and in cohorts B and C. This lapse progressively decreased both in men and women from cohorts A to D. The model including gender, cohort, and interaction between these, significantly predicted the lapse from regular use to AUD onset (F = 9.18, *p* < 0.001), with a significant contribution of cohort (F = 13.15, *p* < 0.001). In the model adjusted for demographic or clinical variables showing significant gender differences (F = 4.99, model *p* < 0.001), the contribution of birth cohort was no longer significant and none of the other variables provided a significant contribution to the model. Similar findings in the lapse from regular use to AUD onset were found by a few studies (see Table 3; [36,68]) while others found no differences in this lapse [27,37,38].

#### 3.2.7. Lapse from AUD Onset to First Request for Care

Among the entire sample, women and men waited for 8.3 and 9.3 years, respectively before the first request for care, with no difference between men and women (see Table 1). This lapse progressively decreased in both men and women from cohorts A to D, with no differences in any birth cohort. The model including gender, cohort and interaction between these, significantly predicted the lapse from AUD onset to first treatment (F = 5.19, *p* < 0.001), with a significant contribution of cohort (F = 3.53, *p* < 0.015). In the model adjusted for demographic or clinical variables showing significant gender differences (F = 3.50, model *p* < 0.001), the contribution of birth cohort was no longer significant. Age at interview was the only variable that provided a significant contribution to the model (F = 4.11, *p =* 0.043). Conversely, other studies found that the lapse from AUD onset to first request of care was shorter in women compared with men with AUD (see Table 3; [5,23,27,29,32,34,36,38,66,67,68]).

## 4. Discussion

Genetic factors have a major role in the development of AUD. Twin and adoption studies have shown that half the risk of developing AUD is heritable [70] and several genome-wide association studies (GWASs) have found significant associations particularly between AUD and genes encoding alcohol-metabolizing enzymes, with significant differences between populations [71]. One example is provided by the mutation observed in approximately 40% of Asian population in the gene encoding the enzyme aldehyde dehydrogenase (ALDH), responsible for the metabolism of alcohol into acetaldehyde [72]. Individuals who are homozygotes for the mutated gene (*ALDH2-2*) are genetically protected against the risk of developing AUD as even low doses of alcohol produce severe nausea and vomiting and an intense skin flush [72]. A series of differences between men and women with AUD in the genetic predisposition to AUD and related phenotypes have been found [73]. For instance, the inactive *ALDH2* genotype has been found to be related to lower age at AUD onset in women with AUD but not in men with AUD; a single nucleotide polymorphism rs27987 (SNP) located in the *GABRA2* gene, encoding the alpha 2 subunit of the GABAA receptor (involved in the inhibitory neurotransmission in the CNS) has been found to increase the risk of developing AUD in men but not in women with AUD [73]. Sardinia has a genetic heritage differing from other contemporary populations, in part explained by selective factors linked to endemic malaria that shaped the genome of its inhabitants and by internal isolation due to the geographic conditions [74]. Accordingly, significant differences would have been expected between Sardinian AUD patients and those living in other countries. However, despite the singular genetic background of Sardinian people [8], low social economic status and levels of education [10], Sardinian AUD patients feature more similarities than differences compared to AUD patients living in other countries or other Italian regions.

In this study, Sardinian men were more frequently affected by substance use disorders than Sardinian women, while women more frequently displayed anxiety and depression and had a family member affected by AUD compared with men. Similar findings were observed in studies conducted in other countries [6,27,32,37,67,68]. The co-occurrence of other mental disorders is frequent among patients with AUD, leading to a worsening of the prognosis of both AUD and other mental disorders and requiring specific interventions [73]. Patients with comorbid AUD and other mental disorders should be referred to specialists in addiction psychiatry [75]. Unfortunately, this type of specialist is not available in Italy. In public healthcare facilities for the treatment of AUD, patients with AUD may also receive medical treatment for substance use disorders from substances other than alcohol. On the other hand, patients with AUD may be referred to other healthcare facilities for the treatment of other mental disorders such as anxiety and/or mood disorders. In other words, men with AUD, more frequently affected than women by additional substance use disorders, may receive treatment for both AUD and other substance use disorders in the same healthcare facility, while women with AUD, more frequently affected than men by anxiety and mood disorders, are often referred to other healthcare facilities for the treatment of these mental disorders. A gender-based approach should take into account these gender differences, introducing the addiction psychiatrist into public healthcare facilities for the treatment of both male and female patients with AUD and other co-occurrent disorders.

Sardinian women were older than men at the age of first drink, regular drinking, and onset of AUD than men, although these differences progressively decreased from the older to younger cohorts. Despite the limited number of women in the oldest and younger cohorts, similar findings were found by studies conducted in other countries [36,38,68], including the progressive reduction in gender differences [5]. No unequivocal results were obtained with regard to progression towards AUD [27,36,37,38,68,69], although the data from this current paper enhance findings indicating how women progress faster than men from regular use to AUD.

Globally, these results suggest that sex and gender differences in drinking characteristics are narrowing among Sardinian AUD patients, as already observed in previous studies, irrespective of differences in genetic make-up and low social economic status and levels of education. However, due to the limited statistical strength in the oldest and youngest birth cohorts, other potential gender differences in these cohorts might not have been detected.

Certain differences in drinking characteristics have also been described between Italian regions. For instance, a remarkable difference was found in the ratio of male/female drinkers between a village in Sardinia (46 men/1 woman) and two villages in Tuscany 3 men/1 woman; [76]. This result may be due to stronger cultural pressure to avoid alcohol being placed on females rather than males in Sardinia than in Tuscany [76]. However, other studies failed to reveal similar differences between Sardinian male and female university students [77,78], suggesting that, both in Sardinia and other Italian regions, cultural pressure to avoid alcohol by females may be decreasing [58].

In this study, the age at first request for care and the lapse from AUD onset to first request for care did not diverge between men and women, while other studies highlighted the younger age of women compared with men at first request for care [27], with women waiting less before seeking medical treatments [5,23,34,36,38,66,67]. These divergences may be related to differences in mean ages of participants, setting, organization of treatment services, cultural and social factors.

The lapse from AUD onset to first request for care progressively decreased from the older to younger cohorts among this study’s Sardinian AUD patients. A similar progressive reduction was observed by a large US study [5]. Nevertheless, the current results demonstrate that Sardinian AUD patients continue to wait a very long time before receiving medical treatment. Shortening this lapse may significantly reduce the negative consequences related to AUD [79].

To improve access and reduce latency in AUD treatment, it would be useful to know the current number of Italian patients with AUD and potential differences between AUD patients who receive treatment and those who do not. Indeed, according to the Italian Ministry of Health, the current number of Italian patients with AUD is still lacking [58], and to the authors’ knowledge, no study to date has investigated the differences between Italian AUD patients who receive or fail to receive treatment, and potential sex and gender differences in these characteristics. A gender-based approach in research in this field should be adopted and studies conducted using appropriate numbers of female and male AUD individuals to further knowledge on these topics.

In view of the increasing number of female AUD patients requiring medical treatment for this disorder, services for treatment of AUD should take into account the specific needs of women. Nonetheless, despite the well-known sex and gender differences in the pharmacokinetics and negative consequences of alcohol [80,81] a sex-gender based approach in the field of AUD is still in its infancy and women’s needs in the treatment of AUD have been completely overlooked. As an example, although women would prefer to receive AUD treatment in sex-and gender-segregated settings, with facilities for childcare, this kind of service is scarce [61]. To facilitate the access of women to medical treatment of AUD, it would be useful to investigate the characteristics of the facilities available in Italy for the treatment of AUD to establish whether women’s needs have been considered and, if so, what actions have been taken, and investigate other possible actions needed to meet their needs.

Although effective medical treatments are available [82,83], AUD is usually underdiagnosed and undertreated [57], with stigma being one of the main reasons for not seeking medical treatment, and a cause of inequalities in health and barriers to treatment [84,85,86]. Women usually experience more severe barriers to AUD treatment than men [61]. It may be the case that only patients with more severe AUD are able to overcome these barriers and gain access to medical treatment. In the present study, patient barriers were not investigated. However, no gender differences were detected in the severity of AUD amongst the sample of AUD patients (see Table 1), with a high mean number of positive criteria in both genders. This may have constituted a sort of “ceiling effect” that might have prevented potential gender differences among patients with less severe AUD from being revealed. Anti-stigma programs should be set up to reduce public stigma and discrimination of patients with AUD and to overcome barriers to treatment for both women and men with AUD.

Additionally, medications used in the treatment of AUD have been studied almost exclusively in male animals and men [80,87], despite the finding of numerous sex and gender differences in response to pharmacological treatments [83]. To improve women’s health, sex and gender should be considered in the treatment of diseases, including AUD [88].

In interpreting this paper’s findings, the following limitations should be considered. Firstly, data were self-reported and may therefore have been influenced by poor recollection or recall bias and under-reporting [89] and by gender differences in reporting [90]. The data were, however, collected by expert psychologists in face-to-face interviews and participants were assured of confidentiality, with no penalties being associated with the reporting of high levels of alcohol consumption.

In conclusion, the present study provided evidence that, despite its specific genetic features, the drinking characteristics of women with AUD in Sardinia, are changing and becoming more similar to those of men, as already observed in other countries. Latency in access to services is still high. In view of the increasing number of women requiring medical treatment for this disorder, more effective policies and improved services are urgently required, particularly relating to female AUD patients; the length of latency moreover should be reduced to allow earlier access to services for patients of all genders affected by AUD.

## Figures and Tables

**Table 1:** Main gender differences and similarities of a sample of Sardinian AUD patients.

	Women	Men	*p*
*n* (%)	66 (19.0)	282 (81.0)	-
Mean age in years (SD)	50.5 (9.0)	47.6 (9.5)	**0.02**
Civil status			
Single *n* (%)	15 (22.7)	134 (47.5)	**<0.01**
Married *n* (%)	24 (36.4)	88 (31.2)	0.46
Separated or widowed *n* (%)	27 (40.9)	60 (21.3)	**<0.01**
Education			
≤8 years *n* (%)	35 (53.0)	178 (63.1)	0.13
>8 years *n* (%)	31 (47.0)	104 (36.9)	0.13
Employment status			
Employed *n* (%)	38 (57.6)	159 (56.4)	0.89
Unemployed *n* (%)	15 (22.7)	68 (24.1)	0.87
Retired *n* (%)	13 (19.7)	55 (19.5)	1.00
Family history			
Father with AUD *n* (%)	21 (36.4)	65 (23.0)	0.15
Mother with AUD *n* (%)	11 (16.7)	6 (2.1)	**<0.01**
Spouse with AUD *n* (%)	8 (12.1)	6 (2.1)	**<0.01**
Comorbidity			
Anxiety disorders *n* (%)	59 (89.4)	199 (70.6)	**<0.01**
Depression *n* (%)	56 (84.8)	174 (61.7)	**<0.01**
Substance Use Disorders *n* (%)	13 (19.7)	114 (40.4)	**<0.01**
Smokers *n* (%)	41 (62.1)	209 (74.1)	0.07
Drinking characteristics			
Severity of AUD			
DSM-IV + criteria for alcohol dependence	5.8 (1.5)	5.7 (1.7)	0.90
Age at first drink in years	16.8 (8.2)	13.7 (4.8)	**<0.01**
Age at onset of regular drinking in years	26.0 (10.2)	19.7 (6.4)	**<0.01**
Age at onset of AUD in years	35.8 (11.1)	31.8 (10.8)	**<0.01**
Age at first request for care in years	44.1 (10.0)	41.1 (10.2)	0.07
Lapse from regular use to AUD onset	9.9 (10.7)	12.2 (10.3)	**0.03**
Lapse from AUD onset to first request for care	8.3 (7.8)	9.3 (9.3)	0.50
Alcohol consumption the year before treatment			
Number of drinks per drinking day	13.0 (9.5)	19.5 (18.1)	**<0.01**
Number of drunk days per year	187.5 (162.4)	186.2 (164.8)	0.76
Severe medical consequences the year before treatment			
Patients with at least 1 ER admission *n* (%)	20 (30.3)	88 (31.2)	0.89
Patients with at least 1 suicide attempt *n* (%)	13 (19.7)	23 (8.2)	**<0.01**

Values are expressed in mean (SD) unless indicated; AUD: Alcohol Use Disorder; DSM-IV + criteria: number of positive criteria for the diagnosis of alcohol dependence of the Fourth Edition of the Diagnostic and Statistical Manual of Mental Disorders (DSM-IV-TR); ER: Emergency Room; SD: standard deviation. Significant differences are reported in bold.

**Table 2:** Gender differences and similarities in drinking characteristics of a sample of Sardinian AUD patients divided in birth cohorts.

Cohort	Number		Age at First Drink (Years)				Age at Onset of Regular Drinking (Years)				Age at Onset of AUD (Years)			
	♀	♂	♀	♂	Δ	*p*	♀	♂	Δ	*p*	♀	♂	Δ	*p*
A (1937–1953)	13	33	14.8 (6.6–22.9)	14.5 (12.5–16.5)	0.3	0.372	28.2 (20.7–35.7)	20.6 (18.1–23.1)	7.6	0.080	48.6 (42.9–54.4)	38.2 (32.9–43.5)	10.5	**0.023**
B (1954–1963)	24	107	18.8 (15.5–22.0)	13.4 (12.5–14.4)	5.3	**0.002**	27.4 (23.0–31.8)	20.6 (19.2–22.0)	6.8	**0.001**	36.6 (33.0–40.3)	35.5 (33.5–37.5)	1.1	0.612
C (1964–1973)	25	94	16.4 (14.3–18.4)	14.2 (13.2–15.2)	2.2	**0.017**	24.4 (20.7–28.1)	19.6 (18.3–20.9)	4.8	**0.008**	30.5 (27.1–33.8)	29.7 (28.1–31.4)	0.7	0.717
D (1974–1991)	4	48	14.0 (5.4–22.6)	13.0 (12.0–14.0)	1.0	0.277	19.8 (9.2–30.3)	17.0 (16.0–18.0)	2.8	0.489	23.0 (11.9–34.1)	23.3 (21.6–25.0)	−0.3	0.916
A (1937–1953)	13	33	58.4 (54.5–62.3)	53.4 (49.8–56.9)	5.0	0.089	20.4 (11.9–28.9)	17.5 (12.3–22.8)	2.9	0.547	9.8 (4.2–15.4)	15.4 (10.6–20.2)	−5.6	0.387
B (1954–1963)	24	107	44.6 (42.7–47.0)	46.5 (45.3–47.7)	−1.9	0.128	9.3 (5.1–13.4)	14.9 (12.8–17.0)	−5.6	**0.010**	8.0 (4.7–11.2)	11.0 (9.1–12.8)	−3.0	0.113
C (1964–1973)	25	94	38.7 (36.3–41.1)	37.0 (35.8–38.3)	1.7	0.265	6.1 (3.6–8.6)	10.2 (8.6–11.7)	−4.1	**0.013**	8.2 (4.9–11.5)	7.3 (5.8–8.8)	0.9	0.734
D (1974–1991)	4	48	28.8 (20.2–37.3)	28.9 (27.4–30.3)	−0.1	0.891	3.3 (1.3–5.3)	6.4 (5.1–7.7)	−3.1	0.126	5.8 (1.0–10.5)	5.4 (3.9–6.8)	0.4	0.492

Values are expressed as mean (95% CI); ♀: women; ♂: men; AUD: alcohol use disorder; Δ: difference between women and men; Δ > 0.0 indicates that women have a higher mean compared with men; Δ < 0.0 indicates that women have a lower mean compared with men. Significant differences are reported in bold.

**Table 3:** Gender differences in drinking characteristics of AUD Sardinian patients compared with those of AUD patients living in other countries.

Reference	*N*	Age in Years Mean (SD)	Civil Status (%)	Familial History (%)	Comorbidity (%)	Age at First Drink in Years Mean (SD)	Age at Onset of Regular Use in Years Mean (SD)	Age at Onset of AUD in Years Mean (SD)	Age at First Request for Care in Years Mean (SD)	Lapse from Regular Use to AUD Onset in Years Mean (SD)	Lapse from AUD Onset to First Request of Care in Years Mean (SD)	g/day Mean (SD)	DSM-IV Criteria for AD Mean (SD)
Agabio et al., Outpatient Sardinia, Italy	66 ♀: 282 ♂	♀: 50.5 (9.0) ♂: 47.6 (9.5) ***p =* 0.02**	Single ♀: 22.7 ♂: 47.5 ***p* < 0.01**	Mother with AUD ♀: 16.7; ♂: 2.1 Spouse with AUD ♀: 12.1; ♂: 2.1 ***p* < 0.01**	Anxiety ♀: 89.4 ♂: 70.6 SUDs ♀: 19.7 ♂: 40.4 ***p* < 0.01**	♀: 16.8 (8.2) ♂: 13.7 (4.8) ***p* < 0.01**	♀: 26.0 (10.2) ♂: 19.7 (6.4) ***p* < 0.01**	♀: 35.8 (11.1) ♂: 31.8 (10.8) ***p* < 0.01**	♀: 44.1 (10.0) ♂: 41.1 (10.2) *p =* 0.07	♀: 9.9 (10.7) ♂: 12.2 (10.3) ***p =* 0.03**	♀: 8.3 (7.8) ♂: 9.3 (9.3) *p =* 0.50	♀: 156 (114) ♂: 234 (217.2) ***p* < 0.01**	♀: 5.8 (1.5) ♂: 5.7 (1.7) *p =* 0.90
Alvanzo et al., 2014 Alcohol-related services US [34]	3311 ♂ and ♀ age < 45 years	NR	NR	NR	NR	NR	NR	NR	♀: 23.1 (22.0–24.3) ♂: 24.9 (24.2–25.6) ***p* < 0.05**	NR	♀: 7.6 (6.6–8.6) ♂: 9.3 (8.6–10.0) ***p* < 0.05**	NR	NR
Ashley et al., 1977 Inpatient Canada [66]	85 ♀ 630 ♂	♀: 44.8 (10.2) ♂: 45.8 (10.2) *p* > 0.05	NR	NR	NR	♀: 18.7 (4.3) ♂: 17.2 (3.7) ***p* < 0.01**	NR	♀: 30.5 (10.5) ♂: 25.4 (8.5) ***p* < 0.01**	♀: 40.5 (9.5) ♂: 39.9 (9.1) *p* > 0.05	NR	♀: 14.1 (8.5) ♂: 20.2 (9.3) ***p* < 0.01**	♀: 227.6 (103.5) ♂: 316 (119.7) ***p* < 0.01**	NR
Blankfield, 1990. In and outpatient Australia [38]	52 ♀ 104 ♂	♀: 45 (NR) ♂: 44.0 (NR) *p =* NR	Single ♀: 18 ♂: 27 *p =* NR	Positive parental history ♀: 37.9; ♂: 40.2 *p* > 0.05	NR	♀: 19.3 (NR) ♂: 15.0 (NR) *p =* NR	♀: 27.5 (NR) ♂: 19.3 (NR) ***p* < 0.001**	♀: 35.0 (NR) ♂: 31.5 (NR) *p =* NR	NR	♀:16.1 (NR) ♂: 24.0 (NR) *p =* NR	♀: 8.5 (NR) ♂: 11.6 (NR) ***p* <** **0.001**	♀: 210 (NR) ♂: 279 (NR) ***p* < 0.001**	NR
Dahlgren, 1978 Inpatients Sweden [68]	100 ♀ 100 ♂	♀: 40.5 (11.9) ♂: 41.0 (11.3) *p* > 0.05	Single ♀: 22 ♂: 25 *p =* NR	Mother with AUD ♀: 4; ♂: 5 *p* > 0.05 Spouse with AUD ♀: 51; ♂: 13 ***p* < 0.001**	NR	♀: 21.2 (5.8) ♂: 17.0 (2.3) ***p* < 0.001**	♀: 27.2 (8.5) ♂: 23.1 (6.6) *p* < 0.001	♀: 34.9 (11.2) ♂: 30.8 (8.8) ***p* < 0.01**	NR	♀: 6.0 (5.6) ♂: 8.0 (5.7) ***p* <** **0.01**	♀: 5.9 (4.7) ♂: 8.2 (5.7) ***p* <** **0.05**	NR	NR
Diehl et al., 2007 Inpatient Germany [36]	106 ♀ 106 ♂	♀: 41.6 (8.4) ♂: 41.9 (8.6) *p* > 0.05	NR	NR	NR	♀: 15.8 (4.4) ♂: 14.5 (4.0) ***p =* 0.02**	♀: 25.3 (7.8) ♂: 20.1 (4.7) ***p* < 0.001**	♀: 35.4 (8.6) ♂: 31.7 (8.5) ***p =* 0.01**	Inpatient treatment ♀: 39.9 (8.6) ♂: 39.6 (9.3) *p* > 0.05	♀: 10.0 (4.1) ♂: 11.6 (4.2) ***p =* 0.047**	Inpatient treatment ♀: 4.5 (4.8) ♂: 7.9 (6.7) ***p* < 0.001**	♀: 153.0 (78.6) ♂:182.8 (103.7) ***p =* 0.02**	NR
Dunne et al., 1993 Unclear UK [37]	121 ♀ 121 ♂	♀: 42.2 (10.1) ♂: 42.5 (9.3) *p* > 0.05	Single ♀: 13 ♂: 16 *p* NR	Positive parental history ♀: 35; ♂: 13 ***p* < 0.05**	Anxiety ♀: 46 ♂: 22 *p* < 0.001 Depression ♀: 37 ♂: 14 ***p* < 0.01**	NR	Median (CI) ♀: 25 (12–61) ♂: 19 (12–55) *p* NR	NR	NR	♀: 9.2 (NR) ♂: 9.6 (NR) *p* > 0.05	NR	♀: 201.1 (NR) ♂: 301.7 (NR) *p* NR	NR
Hernandez-Avila et al., 2004 In and outpatient US [23]	41 ♀ 42 ♂	NR	NR	NR	NR	NR	♀: 20.3 (7.1) ♂: 18.0 (6.4) *p* > 0.05	♀: 20.6 (6.9) ♂: 19.0 (9.6) *p* > 0.05	NR	NR	♀: NR (NR) ♂: NR (NR) ***p* < 0.01**	NR *p* > 0.05	NR *p* > 0.05
Hesselbrock et al., 1985 Inpatient US [67]	90 ♀ 231 ♂	♀: 37.3 (11.0) ♂: 39.5 (12.0) *p* > 0.05	Single ♀: 22 ♂: 30 *p =* NR	NR	Depression ♀: 52 ♂: 32 SUDs ♀: 38 ♂: 45 *p* NR	♀: 15.4 (NR) ♂: 14.1 (NR) ***p* < 0.05**	NR	♀: 30.0 (NR) ♂: 31.5 (NR) *p* > 0.05	♀: 33.1 (NR) ♂: 35.6 (NR) *p =* 0.07	NR	♀: 7.4 (11.0) ♂: 15.0 (12.0) ***p* < 0.05**	NR	NR
Holdcraft and Iacono, 2002a Earlier-born cohort Data NR US [24]	37 ♀ 261 ♂	♀: 44.1 (3.3) ♂: 46.5 (4.5) ***p* < 0.001**	NR	NR	NR	NR	NR	♀: 20.5 (4.5) ♂: 18.6 (3.6) ***p* < 0.05**	NR	NR	NR	NR	♀: 4.4 (1.6) ♂: 4.8 (1.7) *p* > 0.05
Holdcraft and Iacono, 2002b Later-born cohort community-based sample US [24]	95 ♀ 207♂	♀: 36.2 (3.5) ♂: 37.6 (3.6) ***p* < 0.01**	NR	NR	NR	NR	NR	♀:17.0 (3.1) ♂: 17.5 (2.6) *p* > 0.05	NR	NR	NR	NR	♀: 4.5 (1.6) ♂: 4.8 (1.7) *p* > 0.05
Khan et al., 2013 National survey US [6]	1807 ♀ 2974 ♂	NR	Single ♀: 27.6 ♂: 29.5 *p* > 0.05	Positive parental history ♀: 60.2 ♂: 50.5 *p* < 0.05 Spouse with AUD ♀: 38.8 ♂: 13.3 ***p* < 0.05**	Depression ♀: 33.1 ♂: 16.8 Anxiety ♀: 44.3 ♂: 26.2 SUDs ♀: 62.6 ♂: 64.3 ***p* < 0.05**	♀: 17.4 (17.1–17.7) ♂: 16.6 (16.4–16.7) ***p* < 0.0001**	NR	♀: 24.4 (23.8–24.9) ♂: 23.9 (23. 5–24.3) *p =* 0.1402	NR	NR	NR	NR	NR
Keyes et al., 2010a US Data of 2 national surveys Total sample [5]	30.125 ♀ 23.113 ♂	NR	NR	NR	NR	NR	Age at initiation of use ♀: 19.0 (0.04 SE) ♂: 17.8 (0.03 SE) ***p* < 0.001**	NR	NR	From use to AUD ♀: 5.6 (0.15 SE) ♂: 5.8 (0.12 SE) *p* > 0.05	♀: 6.1 (0.4 SE) ♂: 7 (0.3 SE) ***p* < 0.05**	NR	NR
Keyes et al., 2010b US Born 1974–1983 [5]	3292 ♀ 2553 ♂	NR	NR	NR	NR	NR	Age at initiation of use ♀: 17.5 (0.09 SE) ♂: 17.1 (0.08 SE) ***p* < 0.001**	NR	NR	From use to AUD ♀: 3.1 (0.17 SE) ♂: 2.8 (0.12 SE) *p* > 0.05	♀: 5.1 (0.4 SE) ♂: 5.4 (0.4 SE) *p* > 0.05	NR	NR
Keyes et al., 2010c US Born 1964–1973 [5]	7.759 ♀ 5.796 ♂	NR	NR	NR	NR	NR	Age at initiation of use ♀: 18.5 (0.06 SE) ♂: 17.5 (0.05 SE) ***p* < 0.001**	NR	NR	From use to AUD ♀: 3.7 (0.16 SE) ♂: 4.2 (0.16 SE) ***p* < 0.05**	♀: 8.1 (0.6 SE) ♂: 8.1 (0.4 SE) *p* > 0.05	NR	NR
Keyes et al., 2010d US Born 1954–1963 [5]	9.291 ♀ 7.038 ♂	NR	NR	NR	NR	NR	Age at initiation of use ♀: 20.5 (0.09 SE) ♂: 18.4 (0.06 SE) ***p* < 0.001**	NR	NR	From use to AUD ♀: 5.5 (0.31 SE) ♂: 6.4 (0.21 SE) *p* > 0.05	♀: 14.1 (0.5 SE) ♂: 14.8 (0.7 SE) *p* > 0.05	NR	NR
Keyes et al., 2010e US Born 1944–1953 [5]	7.209 ♀ 5.697 ♂	NR	NR	NR	NR	NR	Age at initiation of use ♀: 22.1 (0.22 SE) ♂: 19.1 (0.12 SE) ***p* < 0.001**	NR	NR	From use to AUD ♀: 8.6 (0.44 SE) ♂: 7.9 (0.28 SE) *p* > 0.05	♀: 18.5 (1.1 SE) ♂: 20.0 (0.6 SE) *p* > 0.05	NR	NR
Keyes et al., 2010f US Born 1934–1943 [5]	2.574 ♀ 2.029 ♂	NR	NR	NR	NR	NR	NR	NR	NR	From use to AUD ♀: 11.8 (0.87 SE) ♂: 10.4 (0.63 SE) *p* > 0.05	♀: 19.4 (1.4 SE) ♂: 23.5 (0.9 SE) ***p* < 0.001**	NR	NR
Lewis and Nixon, 2014 Inpatient US [27]	257 ♀ 274 ♂	♀: 33.1 (9.9) ♂: 38.5 (11.1) ***p* < 0.001**	NR	Positive parental history ♀: 73.1 ♂: 60.6 *P* < 0.05 Spouse with AUD ♀: 65.2 ♂: 25.0 ***p* < 0.05**	NR	♀: 12.6 (4.6) ♂: 11.5 (3.9) ***p =* 0.007**	♀: 18.8 (6.0) ♂: 17.6 (4.1) *p* > 0.05	♀: 21.1 (7.6) ♂: 21.5 (8.4) *p* > 0.05	♀: 31.6 (9.2) ♂: 35.1 (10.1) ***p =* 0.007**	♀: 3.1 (4.9) ♂: 4.5 (6.2) *p* > 0.05	♀: 10.3 (9.0) ♂: 14.5 (9.8) ***p* < 0.05**	NR	NR
Piazza et al., 1989 Outpatient and self-help groups US [29]	33 ♀ 105 ♂	♀: 59.3 (8.9) ♂: 63.4 (8.8) NR	NR	NR	NR	♀: 17.5 (2.2) ♂: 15.7 (4.1) ***p =* 0.002**	NR	♀: 34.3 (11.3) ♂: 32.1 (14.7) *p* > 0.05	♀: 43.7 (12.7) ♂: 45.8 (15.3) *p* > 0.05	NR	♀: 10.4 (7.9) ♂: 14.7 (12.6) ***p =* 0.017**	NR	NR
Picci et al., 2012 Inpatient Turin, Italy [69]	56 ♀ 150 ♂	♀: 45.4 (12.1) ♂: 46.3 (10.1) ***p* < 0.05**	Single ♀: 23.2 ♂: 37.3 ***p* < 0.05**	Positive parental history ♀: 62.5 ♂: 45.6 ***p* < 0.05**	SUDs ♀: 23.2 ♂: 37.3 ***p* < 0.05**	NR	♀: 25.9 (11.3) ♂: 21.6 (8.3) ***p* < 0.05**	♀: 34.7 (11.4) ♂: 36.3 (11.0) ***p* < 0.05**	NR	♀: 9 (NR) ♂: 15 (NR) ***p* < 0.05**	NR	NR	NR
Randall et al., 1999 Outpatient US [30]	231 ♀ 634 ♂	♀: 38.3 (10.7) ♂: 38.5 (10.5) *p* > 0.05	NR	NR	NR	NR	NR for outpatients	NR for outpatients	NR for outpatients	NR for outpatients	NR for outpatients	NR for outpatients	DSM III (max 9) ♀: 5.9 (1.8) ♂: 5.9 (1.9) *p* > 0.05
Randall et al., 1999 Outpatient US entire sample [30]	1589 ♀ 1210 ♂	NR for the entire sample	NR	NR	NR	NR	♀: 26.6 (10.3) ♂: 22.7 (9.0) ***p* < 0.001**	Worst problems ♀: 35.2 (10.9) ♂: 35.0 (10.9) *p* > 0.05	NR for the entire sample	NR for the entire sample	NR for the entire sample	NR for the entire sample	NR for the entire sample
Schuckit et al., 1995 Probants for COGA study US [31]	161 ♀ 317 ♂	37.8 (12.6) NR ♀ and ♂	NR	NR	NR	NR	♀: 18.5 (5.3) ♂: 17.5 (4.8) ***p =* 0.04**	NR	♀: 30.1 (8.9) ♂: 32.5 (11.7) *p =* 0.08	NR	NR	NR	NR
Schuckit et al., 1998 Probants for COGA study US 1 drink = 14 g [32]	1.085♀ 2.120 ♂	♀: 36.9 (10.8) ♂: 39.3 (12.7) ***p* < 0.001**	Single ♀: 26.9 ♂: 33.3 ***p* < 0.001**	NR	♀ > ♂ Mood and anxiety ♀ < ♂ SUDs	NR	♀: 18.5 (6.5) ♂: 17.0 (4.2) ***p* < 0.001**	♀: 25.2 (9.3) ♂: 24.2 (8.5) ***p* < 0.001**	♀: 31.0 (9.5) ♂: 31.7 (10.7) ***p* < 0.001**	NR	♀: 3.8 (6.0) ♂: 6.4 (8.1) ***p =* 0.01**	Maximum amount per day ♀: 280 (221.2) ♂: 446.6 (315) ***p* < 0.01**	NR

♀: women; ♂: men; AUD: alcohol use disorder; NR: not reported; SD: standard deviation. In bold: significant gender differences.

## Data Availability

The data that support the findings of this study are available on request from the corresponding author (R.A.).
